# Molecular docking analysis of capsaicin with apoptotic proteins

**DOI:** 10.6026/97320630016555

**Published:** 2020-07-31

**Authors:** Anitha Roy, Arun Rasheed, Aneymol Varikalam Sleeba, Ponnulakshmi Rajagopal

**Affiliations:** 1 Department of Pharmacology, Saveetha Dental College and Hospital, Saveetha Institute of Medical and Technical Sciences, Chennai, Tamil Nadu, India; 2 Department of Pharmaceutical Chemistry, Alshifa College of Pharmacy, Malappuram, Kerala; 3 Department of Microbiology, St. Xavier's College for Women, Aluva, Kerala-683101, India; 4 Central Research Laboratory, Meenakshi Academy of Higher Education and Research (Deemed to be University), Chennai-600 078, India

**Keywords:** Capsicum annum, oral cancer, molecular docking, ADME

## Abstract

Oral cancer is linked with apoptotic proteins such as Bcl-xl, Bcl-2 and Mcl-1. Therefore it is of interest to document the molecular docking analysis of capsaicin (principle present in the Capsicum annum) with apoptotic proteins in this context. We report the molecular binding features of capsaicin with apoptotic proteins such as Bcl-xl, Bcl-2 and Mcl-1 for further consideration.

## Background

Oral cancer is one of the rapidly developing serious issue in most parts of the world. The age depend occurrence of oral cancer may differ from 20 per 100,000 population in India [1]. The external factors include tobacco, chemicals, radiation and infectious organisms and the internal factors include inherited mutations, hormones and immune status causing cancers [2]. The identification of molecular mechanism of oral cancer must be giving extra attention rather. Apoptosis is an important process in embryonic and tissue homeostasis. There are three primary steps occurs in apoptosis i.e initiation, commitment and execution [3]. There are two types of apoptosis proteins i.e pro-apoptotic proteins (Bax and Bak) and anti-apoptotic proteins (Bcl-2, Bcl-xL, Bcl-W, Mcl-1). Pro-apoptotic proteins are important factors for induction of apoptosis through mitochondrial outer-membrane permeabilization, leading to the release of cytochrome c, follow-on in apoptosis by the activation of caspases. The normal apoptosis process need the normal level of balance between the -apoptotic and pro- apoptotic proteins [4] in cancer, there is an imbalance in this ratio as many anti-apoptotic proteins are available to inhibit apoptosis by forming heterodimers with pro-apoptotic proteins and thus inactivate them [5]. So identification of molecules that supports apoptosis by targeting both intrinsic and extrinsic apoptotic pathways was helped to understand the mechanism behind tumor cell proliferation, which can leads to the development of efficient cancer therapies. Recently, phytocompounds have gained prominence, as they can alter the cell cycle, apoptosis evasion, angiogenesis and metastases. They have confirmed their efficiency in mono treatments or in combination with other chemo preventive agents. Capsicum annuum or Red chili spur pepper (hot pepper), is one of the frequently used spice in Thai cuisine. Capsaicin, the component present in the C. annuum, responsible for the spiciness of chili peppers. Capsaicin is an alkaloid compound used in food, spices and medicines worldwide [6-8]. It is used to treat muscular pain and headaches. It is also used to improve circulation, for its gastrointestinal protecting effects; and to fight against many types of cancer [9, 10].

## Materials and Methods:

### Structures of Bcl-xl, Bcl-2 and Mcl-1:

Three-dimensional structures of apoptotic proteins Bcl-xl (PDB id: 2O1Y), Bcl-2, (PDB id: 4MAN), Mcl-1 (PDB ID: 3D7V) were downloaded from protein data bank (PDB) [11].

### Ligand preparation:

Structure of Capsaicin was retrieved from Pubchem database in SDF format. It was converted as PDB format using HEX software.

### Molecular Docking Analysis:

Hex8.0 software (http://hex.loria.fr)[12] was used for docking studies to identify the possible interaction between Bcl-xl, Bcl-2 and Mcl-1 with Capsaicin.

### Molecular descriptors calculation:

Smiles notation of Capsaicin was helped to calculate the molecular descriptors using Molinspiration (www.molinspiration.com). They molecular descriptors like log P, molecular weight, polar surface area, number of atoms, number of rotatable bond, number of O or N, number of OH or NH, ion channel modulator, drug-likeness and number of violations to Lipinski's rule were calculated in the present study [13].

### Analysis of drug likeness of selected compounds:

The drug likeness property of Capsaicin was estimated with help of Lipinski filter (http://www.scfbio-iitd.res.in/software/ drugdesign/ lipinski.jsp), according to that, the orally active drug must follow minimum four of five laid down criterion for drug likeness namely: molecular mass, cLogP, hydrogen donor and acceptor and molar refractive index [14].

## Results and discussion:

**Figure 1:**
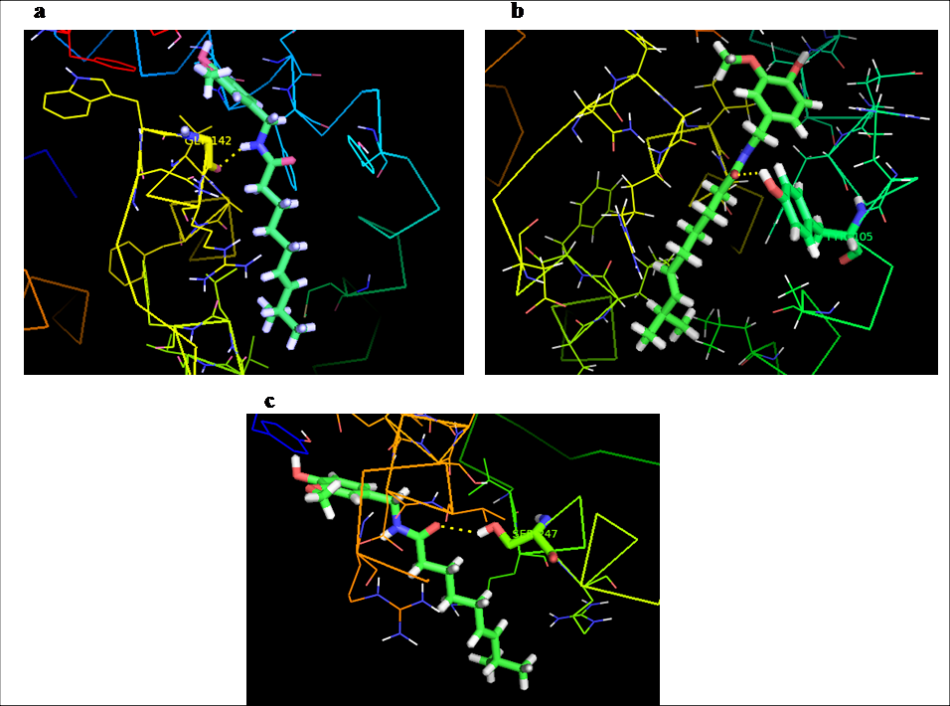
Molecular docking binding features of capsaicin with apoptotic proteins such as (a) Bcl-xl, (b) Bcl-2 and (c) Mcl-1

The molecular docking study we report here identified the binding mode and intermolecular interaction between capsaicin and Bcl-xl, Bcl-2 and Mcl-1 proteins. Identification of interaction energies among the compound and proteins has been a big challenge for molecular docking studies. Hex calculates this binding energy of the docked complexes by using scoring algorithms. The steadiness of these complexes will be high if the energy value is low. Higher the negative Etotal value stronger is the interaction between compound and proteins, which lead to activation of proteins. The binding energy was calculated in terms of KJ/mol. Analysis of obtained docking results confirmed that compound Capsaicin strongly binds with the apoptotic proteins because all three proteins (Bcl-xl--283.78, Bcl-2- -258.17 and Mcl-1- -237.50) showed the highest negative energy value when docked with Capsaicin (Table 1). Hence it was confirmed that all complexes were in stable form. PyMol analyses of docked complexes were showed the vital interaction information about the binding sites and their orientation of inhibition in the target proteins. It also showed the interacting amino acids residues between the protein and ligand (Figure 1). This insilco analysis on the activities of Capsaicin based on the docking scores provides exact understanding for compound and protein binding interaction, which can be used for newer drugs against oral cancer.

Prediction of drug-likeness was necessary to pharmacological industries to enhance the activity and selection of compounds. This also decreases the probability choosing the false positive compounds. In the current study drug likeness properties of Capsaicin was calculated through Lipinski filter and the properties of the compound by respect to calculation of adsorption, distribution, metabolism, excretion and toxicity. This tool is really useful in predicting the drug likeness properties of compound (Table 2). Lipinski filter results for Capsaicin have good drug likeness properties, so it can use as drug for application in biological systems. Molinspiration was performed to estimate the properties and drug likeness score of the Capsaicin. Violation of the Lipinski's rule of five is when logA is >5, MW >500, number of N, O (hydrogen bond receptor) is >10, number of OH and NH (hydrogen bond donor) is >5 and number of the rotatable bond (rotb) is >15 (21). As per the range, physiochemical properties capsaicin illustrated zero violation and fulfilled well by the Lipinski's rule of five as shown in (Table 3). Therefore the results of Docking studies and ADME evidently proved that capsaicin have some activity against oral cancer.

## Conclusions:

We report the molecular binding features of capsaicin with apoptotic proteins such as Bcl-xl, Bcl-2 and Mcl-1 for further consideration.

## Figures and Tables

**Table 1:** Molecular docking results obtained from HEX

S.No	Protein name	E.Total	H- bond details	No of non bonded contacts
1	Bcl-x	-283.78	TYR 105 -H	48
2	Mcl-1	-237.5	SER-247 -H	33
3	Bcl-2	-258.17	GLY-142 -O	70

**Table 2:** Calculated ADME Properties

Compound Name	Molecular Mass a	Hydrogen bond donorb	Hydrogen bond donor c	LOGPd	Molar Refractivitye
Capsaicin	305	2	4	3.789599	88.951469
aMolecular mass less than 500 Dalton; bHigh lipophilicity (expressed as LogP less than 5); cLess than 5 hydrogen bond donors; dLess than 10 hydrogen bond acceptors; eMolar refractivity should be between 40-130

**Table 3:** Calculated molecular Descriptors using Mol inspiration

Compound name	miLogPa	TPSAb	natomsc	MW d	nONe	nOHNHf	nviolationsg	nrotbh	volumei
Capsaicin	3.1	58.56	22	305.42	4	2	0	9	310.37
aLogarithm of partition coefficient between n-octanol and water (miLogP); bTopological polar surface area (TPSA); cNumber of hydrogen bond acceptors (n-ON). dNumber of hydrogen bond donors (n-OHNH); eNumber of rotatable bonds (n-rotb); fPercentage of absorption (%ABS); gMolecular weight (MW)
